# All animals are conscious in their own way: comparing the markers hypothesis with the universal consciousness hypothesis

**DOI:** 10.3389/fpsyg.2024.1405394

**Published:** 2024-05-13

**Authors:** Angelica Kaufmann

**Affiliations:** Cognition in Action Lab, University of Milan, Milan, Italy

**Keywords:** animal consciousness, markers hypothesis, dimensions, distribution, animal cognition

## 1 Introduction

Consciousness in non-human animals can be explored philosophically through two central questions: the distribution question, which enquires which animals are conscious, and the phenomenological question, which seeks to understand what the experiences of animals are like (Allen and Trestman, 2024).

The distribution question is considered empirically tractable by those scientists who believe that markers, such as traits or behaviors, can be used to assess the presence of consciousness in animals. Bayne et al. (2024) have recently offered a version of this markers approach. They also aim to make the phenomenological question empirically tractable by targeting phenomenological experience through potential C-tests with the aim of identifying conscious entities across a spectrum of beings, including humans, animals, and artificial systems. Indirectly, Andrews (2024) also advocates for the empirical tractability of the phenomenological question and indirectly criticizes the marker-based approach, highlighting its inadequacies in addressing the “distribution question” of consciousness—namely, which animals are conscious. She argues for a paradigm shift that favors an inclusive presumption that all animals possess consciousness, challenging the premise of needing C-tests to distinguish conscious from non-conscious entities.

Acknowledging the complexity of applying C-tests to non-human entities, Bayne et al. reference Dung and Newen (2023), who propose a species-sensitive, two-tier account of animal consciousness, aiming to assess not just whether animals are conscious (the distribution question) but also how their conscious experiences differ (the phenomenological question). Both approaches highlight the diversity of conscious experiences in the animal kingdom and encourage ethical considerations regarding the treatment of other animal species.

Andrews does not engage with Dung and Newen directly. Her focus is on proposing a foundational shift in how we approach the study of animal consciousness, arguing for the assumption that all animals are conscious as a starting point for research. This approach contrasts with seeking specific markers or dimensions of consciousness, as Bayne et al. and Dung and Newen suggested frameworks do, or Birch et al. (2020) before them, by instead questioning the very methodologies we use to infer consciousness in non-human animals.

Bayne et al. champion the utilization of precise markers, or C-tests, to demarcate conscious entities. Their methodology, underscored by a commitment to scientific rigor, seeks to establish a clear boundary between conscious and non-conscious beings. This approach, whilst promising methodological clarity, may inadvertently overlook the intricate and varied nature of consciousness, potentially imposing anthropocentric limitations on the understanding of animal consciousness. However, Andrews's broad ethical presumption of consciousness across all animals may risk diluting the specificity required to discern the diverse manifestations of consciousness across species.

Each perspective presents its merits—Bayne et al.'s methodological clarity, and Andrews' ethical inclusivity. It is Dung and Newen's account that appears to provide a preferable methodological synthesis where the identification of markers is informed by an ethical commitment to presume consciousness broadly, all whilst acknowledging diversity across species.

## 2 The markers hypothesis

Bayne et al. (2024) introduce the concept of C-tests, emphasizing the urgent need for validated methods to determine consciousness across different systems, including humans at various developmental stages, non-human animals, AI, and more recent innovations such as neural organoids and xenobots. Bayne et al. highlight the general consensus on consciousness in healthy, awake adult humans but acknowledge the debate on the presence of consciousness in other entities or states, such as during human development, in sleep, under anesthesia, and in various brain-damaged conditions. They also point out the controversies over consciousness in non-human animals.

The authors propose a four-dimensional space for classifying potential C-tests. These dimensions include the target population (identify which entities the C-test is applicable to, such as humans, specific animals, or artificial systems), specificity (measure the false-positive rate of the C-test since a test with high specificity accurately indicates consciousness when it is present), sensitivity (the test's ability to correctly identify true positives—genuinely conscious entities), and rational confidence (the degree of trust in the test's specificity and sensitivity assessments). To validate C-tests, Bayne et al. suggest three strategies:

The redeployment strategy: using variants of widely accepted tests for consciousness.

The theory-based strategy: grounding tests in consciousness theories.

The iterative natural kind strategy: an iterative process of refining and validating tests, treating consciousness as a natural kind.

This latter, indicated as the preferred strategy, posits that C-tests should be applied hierarchically, beginning with “consensus cases” (e.g., neurotypical, adult humans) and extending to “neighboring” and then more “alien” populations.

The authors recognize the moral implications of consciousness assessment, especially since consciousness is often linked to moral status (Shepherd, 2018, 2023). They acknowledge the importance of aligning C-tests with ethical considerations, as consciousness may dictate how various entities should be treated.

Bayne et al. also address the challenge of applying these tests to non-human subjects, particularly when certain abilities required by the test may be specific to humans, such as language or certain patterns of neural activity.

The significance of Bayne et al.'s studies lies not only in the advancement of C-tests but also in the broader philosophical and ethical discourse on consciousness. By considering different population targets and validating the sensitivity and specificity of these tests, Bayne et al.'s studies directly contribute to the ongoing dialogue on animal consciousness and how to appropriately measure it.

Bayne et al.'s (2024) proposal exemplify methodological rigor through its systematic and interdisciplinary approach. It sets forth a comprehensive framework to classify tests as C-tests, considering diverse entities from human development to artificial systems. This framework is underpinned by a precise categorization based on the target population, specificity, sensitivity, and rational confidence, each dimension addressing distinct validation challenges. The authors expand the robustness of their approach by critically assessing three validation strategies: redeployment, theory-based, and iterative NK, thus avoiding reliance on a single, potentially narrow methodological pathway. The authors advocate for an iterative NK strategy that emphasizes flexibility and adaptability, allowing for the refinement of hypotheses and methods in light of new evidence. By transparently discussing the inherent limitations and crucial decision points of developing C-tests, the authors exhibit a conscientious understanding of the complexity of their research question. This self-reflective stance not only clarifies the methodological boundaries but also ensures that the research advances with clarity and precision.

Although not directly addressing it, their paper can be understood as a response to Andrews' (2024) view that “all animals are conscious” and challenges it by proposing a structured, methodological framework for assessing consciousness across a broad spectrum of entities. This may sound in contrast with Andrews' position, which promotes an assumption of consciousness across all animals as a foundational starting point for research. Instead, Bayne et al.'s methodology could offer a systematic way to test Andrews' assertion and investigate the dimensions of consciousness she suggests should be the focus of research.

## 3 Universal consciousness

Andrews (2024) advocates for a paradigmatic shift in consciousness studies: the scientific community should adopt the stance that all animals are conscious by default and then work to explore dimensions of consciousness rather than laboring to mark consciousness in different species.

This approach, she argues, is limited by its reliance on initial markers—pretheoretical indicators such as language, social responsiveness, and emotional expression—and its development of derived markers—indicators that emerge from scientific investigation.

Andrews points out that as research progresses, the number of derived markers for consciousness increases, leading to a higher probability of ascribing consciousness to various species, potentially even those such as *Caenorhabditis elegans* and Hydra, which traditionally might not be considered conscious.

Andrews suggests that this approach creates an illusion of progress on the distribution question of consciousness because it can only increase the confidence in an animal's consciousness, not decrease it.

Initial markers are simply characteristics observed that set a baseline for the study of consciousness but are insufficient as proof. For instance, the fact that an entity displays pain behavior or engages in goal-directed activities does not conclusively demonstrate consciousness. This is particularly true in organisms whose physical forms or neural architectures differ significantly from humans or in the case of artificial intelligence. Conversely, derived markers arise through more theoretical means and often reveal aspects of consciousness not immediately evident through initial markers. These can encompass a range of behaviors that pass certain tests, or they can be mechanistic, rooted in the neurophysiology or biochemistry of the entity in question. These markers are less human-centric, recognizing behaviors and structures distinct from those typically found in humans, as long as they fulfill similar functional roles. The derived marker approach accommodates the multiple realizability of psychological properties, indicating a move toward a more inclusive and varied recognition of consciousness markers.

Andrews recommends that scientists default to the assumption that all animals are conscious and then investigate the various expressions and intensities of consciousness. This change in the scientific stance could catalyze more comprehensive and productive research, facilitating the development of a rich and inclusive theory of consciousness built on data spanning a vast array of life forms.

In essence, Andrews' argument is both pragmatic and methodological. She suggests that accepting the premise that all animals are conscious would eliminate biases that could hinder research and would leverage simpler organisms to gain insights into consciousness that might be obfuscated in more complex beings. Embracing this foundational shift would not only enhance the study of animal minds but could also have ethical implications for their treatment, emphasizing the importance of understanding the subjective experiences of non-human beings.

## 4 Between markers and dimensions

Dung and Newen (2023) propose a framework between markers and dimensions by addressing simultaneously the distribution question (which animals are conscious) and the phenomenological question (how consciousness experiences differ between animals).

The framework establishes 10 dimensions of consciousness with species-sensitive operationalizations, which allows for a comprehensive comparison of consciousness profiles across different animal species. This approach differentiates between strong and weak indicators of consciousness, enabling researchers to assign a multi-faceted profile to animal species, reflecting their conscious experiences. Strong indicators are direct evidence of consciousness, whilst weak indicators require multiple instances or higher degrees of the behavior to suggest conscious experience. Dung and Newen build upon previous studies by Birch et al. (2020), whilst making four key advancements in their methodology: (1) a distinction between the distribution and the phenomenological question; (2) a structured taxonomy with strong and weak indicators; (3) the inclusion of dimensions for cognitive processing strategies beyond content features of conscious experience; and (4) a more extensive set of ten dimensions as opposed to the five suggested by Birch et al. (2020). The five dimensions included: perceptual richness (how fine-grained is perception), evaluative richness (how fine-grained is valence), integration at a time (how temporally integrated is an experience), integration across time (how continuous or fragmented is an experience), and self-consciousness (how conscious of being a specific entity separate from the environment). Whereas, Dung and Newen add three dimensions of cognitive processing strategies: complex forms of reasoning (such as transitive inferences and causal reasoning), some forms of learning, and abstract categorization of specific sensory stimuli or events. They also include two further dimensions: the experience of body and mental agency and that of body ownership. The experience of agency pertains to whether an animal perceives its actions, including mental actions, as self-generated and under its voluntary command, rather than as occurrences that exceed their control (such as, mind wandering). The experience of ownership determines whether an animal recognizes its body parts as intrinsic to its being or merely as objects existing within the external environment.

They argue that these 10 dimensions are core for any general investigation of animal consciousness, but they are adaptable for more specific comparisons, such as between two species or different stages of ontogenetic development.

The operationalizations for these dimensions draw from a variety of behaviors and cognitive abilities. For example, perceptual categorization can be measured through tests such as discrimination learning and motivational trade-offs, whereas agency might be gauged through tasks testing delay of gratification or response inhibition.

Their studies contribute to the understanding of animal consciousness by offering a structured framework that can inform both empirical research and ethical considerations about the treatment of animals. Their approach specifically seeks to recognize indicators of consciousness that are potentially unique to non-human animals, which could differ significantly from human consciousness markers. In addition, the introduction of strong and weak indicators adds a layer of complexity to the evaluation of consciousness. This distinction acknowledges that not all indicators provide the same level of evidence for consciousness, and a set of weaker indicators can collectively signal the presence of consciousness in an animal. Process-oriented indicators for cognitive processes such as reasoning, learning, and abstraction reflect a deeper inquiry into how consciousness operates rather than just its outward manifestations. This shows an interest in the mechanisms of consciousness, providing a richer picture than what might be obtained through more static, trait-based markers. A defining feature of their framework is its adaptability and openness to revision based on empirical findings. This flexibility is an acknowledgment of the evolving nature of consciousness science. Their framework is not just theoretical but comes with concrete operationalizations for each dimension, providing tangible, testable manifestations of consciousness. This aspect is particularly valuable as it moves the field beyond theoretical speculation to empirical investigation. Furthermore, the authors recognize the limitations of current methodologies and introduce what they term pragmatic idealizations. This approach is intended to guide and refine research without making unwarranted assertions, which marks a departure from the sometimes binary perspective of traditional markers.

Dung and Newen's perspective can be seen as an intermediary between the marker-based approach of Bayne et al. and the universal consciousness claim argued by Andrews. Whilst they utilize a form of marker through their structured taxonomy, their approach is species-sensitive and acknowledges the diversity and richness of consciousness across species.

## 5 Conclusion

A balanced perspective on animal consciousness requires both empirical and ethical sensitivities. The C-tests proposed by Bayne et al. (2024) bring a necessary scientific precision to the field, whilst Andrews (2024) ethical presumption of universal consciousness ensures the moral consideration of all animals. Dung and Newen (2023) multi-dimensional framework integrates these aspects, offering a methodological approach that is both scientifically informed and ethically aware, incorporating the strengths of each perspective.

## Author contributions

AK: Conceptualization, Methodology, Resources, Writing – original draft, Writing – review & editing.
